# Variable cardiac responses in rhesus macaque monkeys after discrete mediodorsal thalamus manipulations

**DOI:** 10.1038/s41598-023-42752-4

**Published:** 2023-10-07

**Authors:** Juan Carlos Méndez, Brook A. L. Perry, Elsie Premereur, Vassilis Pelekanos, Tamara Ramadan, Anna S. Mitchell

**Affiliations:** 1 Department of Clinical and Biomedical Sciences, University of Exeter, College House, St Luke’s Campus, Heavitree Road, Exeter, EX1 2LU UK; 2 Medical Research Council Brain Network Dynamics Unit, Nuffield Department of Clinical Neurosciences, University of Oxford, Mansfield Road, Oxford, OX1 3TH UK; 3 Laboratory for Neuro- and Psychophysiology, KU Leuven, Leuven, Belgium; 4 School of Medicine, University of Nottingham, Nottingham, UK; 5 Department of Biological Sciences, University of Oxford, Oxford, UK; 6 Department of Psychology, Speech and Hearing, University of Canterbury, Christchurch, 8041 New Zealand

**Keywords:** Neuroscience, Cognitive neuroscience, Diseases of the nervous system, Emotion, Learning and memory, Neuro-vascular interactions, Reward, Physiology

## Abstract

The control of some physiological parameters, such as the heart rate, is known to have a role in cognitive and emotional processes. Cardiac changes are also linked to mental health issues and neurodegeneration. Thus, it is not surprising that many of the brain structures typically associated with cognition and emotion also comprise a circuit—the central automatic network—responsible for the modulation of cardiovascular output. The mediodorsal thalamus (MD) is involved in higher cognitive processes and is also known to be connected to some of the key neural structures that regulate cardiovascular function. However, it is unclear whether the MD has any role in this circuitry. Here, we show that discrete manipulations (microstimulation during anaesthetized functional neuroimaging or localized cytotoxin infusions) to either the magnocellular or the parvocellular MD subdivisions led to observable and variable changes in the heart rate of female and male rhesus macaque monkeys. Considering the central positions that these two MD subdivisions have in frontal cortico-thalamocortical circuits, our findings suggest that MD contributions to autonomic regulation may interact with its identified role in higher cognitive processes, representing an important physiological link between cognition and emotion.

## Introduction

Emotional responses to environmental stimuli involve complex physiological changes that provide the organism with important cues about the salience and value of a stimulus to facilitate appropriate action^1^. A crucial component of these responses is the regulation of cardiovascular activity, including the heart rate (HR), heart rate variability, and blood pressure (BP). Several brain structures form a circuit, known as the central autonomic network (CAN), that modulates HR via the sympathetic and parasympathetic branches of the autonomous nervous system^2^. The anterior cingulate, pregenual cingulate, orbitofrontal, and insular cortices, as well as the precuneus, temporal pole, amygdala, thalamus, hypothalamus, periaqueductal gray, pons, medulla, and cerebellum, have all been suggested to integrate this network^3^. Depending on the environmental challenges, some of these nodes become particularly active and coordinate the appropriate cardiovascular response; for example, the nucleus of the tractus solitarius and nuclei in the ventral medulla are known to regulate the HR in response to blood pressure changes^4^. The mechanisms behind cardiovascular adaptations to more complex stimuli, however, such as mental or emotional stressors, are still incompletely understood.

The importance of better understanding the function that each node in the CAN has in linking emotion, cognition, and HR becomes evident when we consider that cardiovascular disease and mental health, especially depression, are closely linked^5^. The same is true regarding the known correlates between cardiovascular disease, cognitive decline, and neurodegenerative diseases^6^. Historically, the frontal and insular cortices have been given pre-eminence when studying these correlations given that substantially altered cognitive and emotional states, together with abnormal cardiovascular activity, arise with damage to these cortical regions, respectively^7^.

Recent evidence from functional neuroimaging studies in humans^8–10^ and anatomical work in non-human primates (NHPs;^11^) suggest the mediodorsal nucleus of the thalamus (MD) could also be an important node within this cardiac neural network. The MD is reciprocally interconnected to the entire frontal cortex, with its magnocellular (MDmc) and parvocellular (MDpc) subdivisions showing segregated, although partially overlapped connectivity patterns across the frontal cortex, especially in the anterior cingulate cortex^12,13^. Importantly, cortico-thalamocortical connectivity changes, many involving the MD, are now characterized in several neuropsychiatric and neurodegenerative disorders where alterations in cognitive abilities, arousal, and cardiovascular parameters are prominent features, including schizophrenia, frontotemporal dementias, and mood disorders^9,14–16^.

Previous neurophysiological experiments in rats^17^ and rabbits^18,19^ had already pointed out that neuronal activity in the MD correlates with HR changes. However, to our knowledge, these experiments have not been replicated in NHPs or humans. This poses a problem, as behavioral and physiological findings in one species are not necessarily translatable to others^20^. Thus, if we want to confidently transfer experimental findings in animals to human clinical applications, it is crucial that experiments are performed in those species known to have the closest neuroanatomical and physiological similarity^20,21^.

In our lab, we have performed different experimental manipulations of the MDmc and MDpc in NHPs to study their roles in cognition (e.g.,^22–25^). Here we report that, whilst manipulating the MD in these experiments, we observed heart rate and blood pressure changes that suggest that, in addition to its known role in supporting the cortex to compute higher cognitive processes, the MD may also support aspects of autonomic responses, contributing to the creation of an important link between physiology, emotion, and cognition. The observable evidence for heart rate changes presented here occurred while NHPs received either unilateral MDmc or MDpc microstimulation, or unilateral or bilateral cytotoxin injections into the MDmc and/or MDpc to lesion these areas under surgical anaesthesia. For neurosurgical comparison purposes, we also include data from other groups of monkeys undergoing similar neurosurgical and anaesthesia procedures in our lab, but without MD manipulations.

## Results

### Microstimulation of the MD

**Figure 1:**
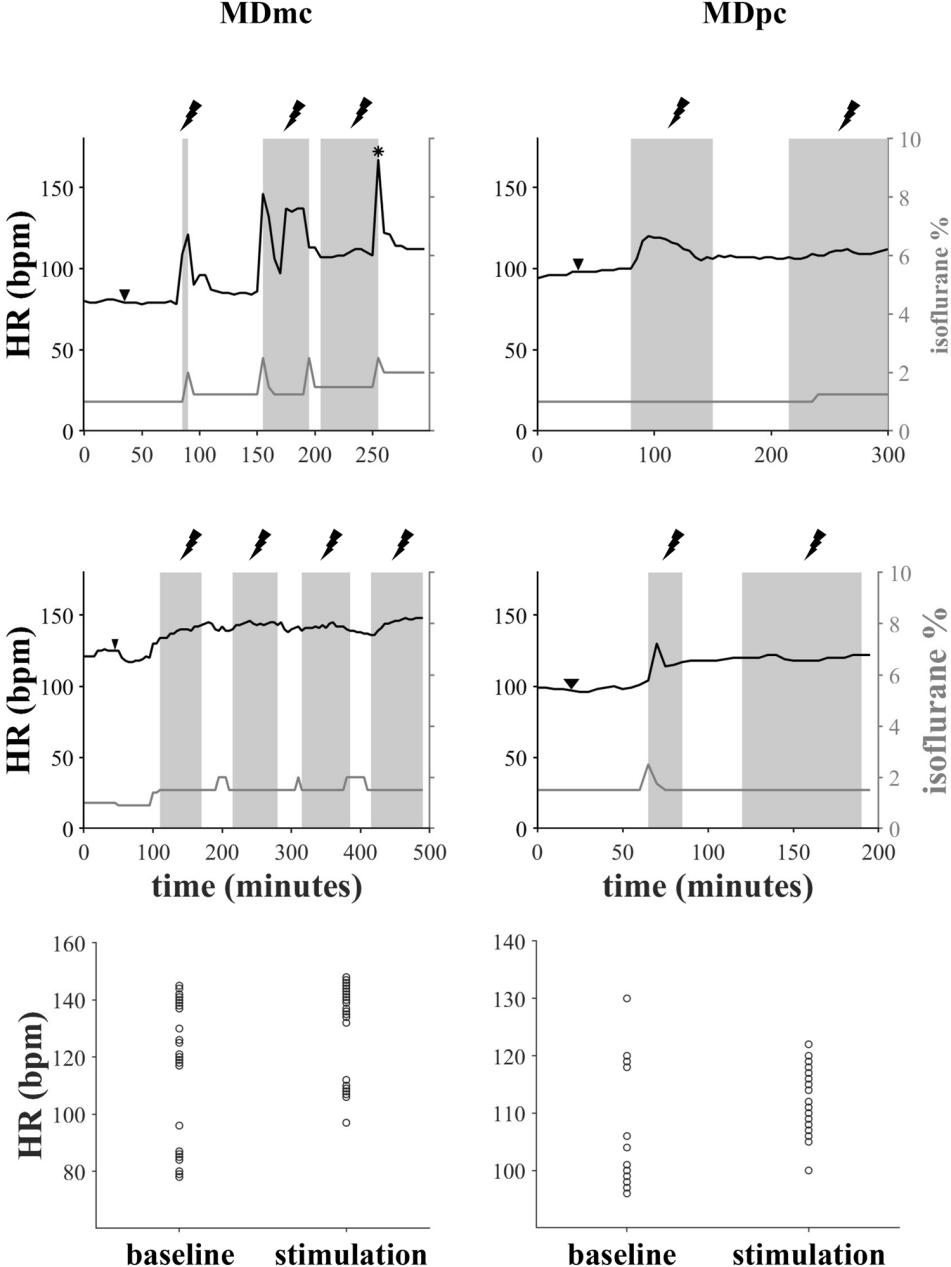
Effect of microstimulation in the magnocellular (MDmc, left column) and parvocellular (MDpc, right column) subdivisions of the mediodorsal thalamus on the heart rate (HR). (Top and middle row) The moment the microelectrode was positioned in the corresponding structure is pointed out with a downward arrowhead. Periods when microstimulation was administered are shown with a grey background. Also shown is the percent concentration of gaseous anaesthesia (isoflurane %) administered throughout the experiment (grey line). The asterisk * in the top left panel indicates a moment when the animal was taken out of the scanner bore and showed a change in muscle tone, likely causing the brief increase in heart rate. (Bottom row**)** Individual HR measurements during the baseline and stimulation periods for each MD subdivision.

In four separate sessions, we microstimulated the right MDmc of two male macaque monkeys and the MDpc of two male macaque monkeys under general anaesthesia (see Materials and Methods). The heart rate during MDmc microstimulation periods was higher than during their corresponding baseline periods (baseline mean = 114.1 bpm, standard deviation (SD) = 23.9; stimulation mean = 135.7 bpm, SD = 13.7; Fig. 1, left column). Equally, the mean arterial pressure was higher during stimulation (mean = 56.1 mmHg, SD = 9.3) than during baseline periods (mean = 42.9 mmHg, SD 4.2). We performed a linear mixed-effects analysis which showed that both the stimulation and the isoflurane levels significantly affected the HR (p < 0.001 for both factors). However, this same analysis showed that only the stimulation (p < 0.001), but not the isoflurane levels (p = 0.17) affected the BP.

In the case of the parvocellular subdivision of the MD, the heart rate and the mean arterial pressure were again higher during stimulation than during baseline (HR baseline mean = 103.5 bpm, SD = 9.4; HR stimulation mean = 113.6 bpm, SD = 5.8; BP baseline mean = 50.6 mmHg, SD = 8.4 ; BP stimulation mean = 51.8 mmHg, SD = 4.4). In this case, the linear mixed-effect model analysis showed that the HR was affected only by stimulation (p < 0.001), but not by isoflurane levels (p = 0.62). However, the BP was affected both by the stimulation (p < 0.001) and isoflurane levels (p = 0.01; Fig. 1, right column).

### Cytotoxin injections and neurosurgical brain manipulations

Initially, we used a one-way ANOVA to compare changes in the mean percent heart rate difference score (see Materials and Methods) between the three different groups of monkeys that received either a cortical ablation of the retrosplenial cortex, the ventral prefrontal and orbitofrontal cortex, or a fornix transection. There was no significant change in mean percent heart rate difference scores between these three groups (*F*(2,12) < 1.0), so their scores were combined to complete all further analyses with these monkeys as the control lesion group.

**Figure 2:**
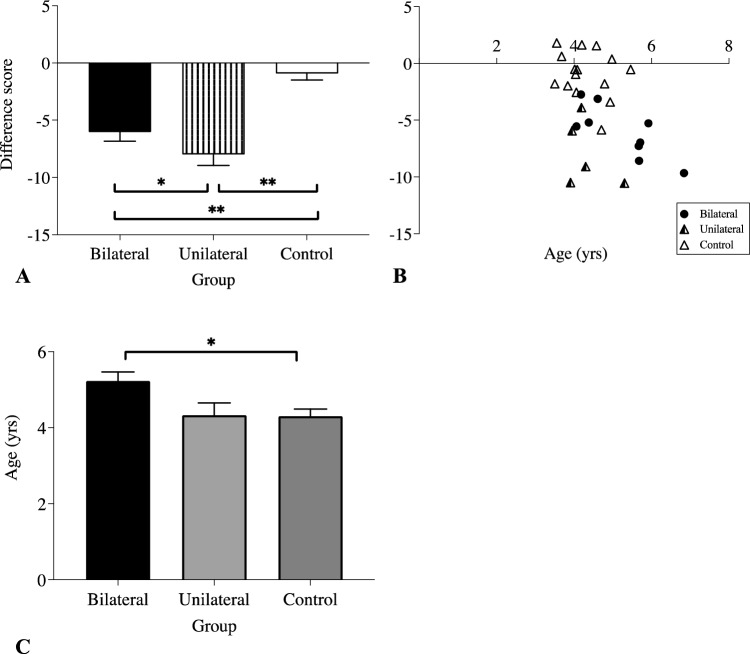
(**A**) Mean percent heart rate change difference scores as a function of brain manipulation type. (**B**) Correlation showing effect of age in years at time of neurosurgery on the mean percent heart rate change difference score. (**C**) Mean age in years at time of neurosurgery for each brain manipulation group. Bilateral: bilateral cytotoxin infusions of NMDA and ibotenic acid to the mediodorsal thalamus (either magnocellular MD (MDmc) or parvocellular MD (MDpc)); Unilateral: unilateral cytotoxin infusions of NMDA and ibotenic acid to the MDmc; Control: bilateral ablations to the retrosplenial cortex, fornix transection, or a combined unilateral ablation to orbitofrontal and ventrolateral prefrontal cortex.

As indicated in the literature^26^, age influences heart rate, and this was evident in our data. A bivariate Pearson’s correlation between monkey’s age and heart rate difference score showed that the monkey’s age at the time of neurosurgery produced a significant negative correlation (r = − 0.469, *p* = 0.009, two-tailed; see Fig. 2B), indicating that older monkeys had a greater change in their heart rate difference score than younger monkeys. However, the partial correlation analysis did not show an effect of age on heart rate change when controlling for brain manipulation (r = − 0.227, *p* = 0.245; see Fig. 2B). Further *posthoc* comparisons of this effect of age indicated that the ages of monkeys in the bilateral MD cytotoxin group at the time of neurosurgery (M = 5.22, SD = 0.96) were older than the control lesion group (M = 4.33, SD = 0.58; *p* = 0.015), but not the unilateral MD cytotoxin group (M = 4.33, SD = 0.57; *p* = 0.102); the comparison of ages between control lesion group and the unilateral MD cytotoxin group was not significant (*p* = 1.0; see Table 1; Fig. 2C).

**Table 1:** Heart rate changes during baseline and after brain manipulations for rhesus macaque monkeys undergoing neurosurgical procedures.

Monkey, sex, and lesion type	Age	Mean HR baseline	Range	Mean HR lesion	Range	% change HR	% change sevoflurane
Bilateral MD lesions including MDmc and/ or MDpc using midline approach							
MDP1* (M) (Bilat MDpc)	6.84	114.8	110–117	103.7	98–107	− 9.66	− 6.8
MDP2* (M) (Unilat MDmc + Unilat MDpc)	4.05	119.5	117–121	112.9	109–118	− 5.55	− 2.1
MDP3* (M) (Unilat MDmc + Unilat MDpc)	4.18	114.8	114–116	111.6	108–114	− 2.74	0.0
MDP4* (M) (Bilat MDmc + Bilat MDpc)	4.38	123.6	122–125	117.2	100–140	− 5.20	11.9
MD1^#^ (M) (Bilat MDmc)	5.66	120.1	119–121	111.4	108–115	− 7.27	− 1.5
MD2^#^ (M) (Bilat MDmc)	4.60	123.6	122–124	119.8	117–121	− 3.12	1.6
MDX^$^ (M) (Bilat MDmc)	5.68	106.8	103–109	97.6	92–100	− 8.58	15.0
MDY^$^ (M) (Bilat MDmc)	5.71	107.8	105–110	100.3	97–105	− 6.96	2.0
MDZ^$^ (M) (Bilat MDmc)	5.92	116.1	116–117	110.0	106–114	− 5.27	7.0
Unilateral MDmc/midline lesion using midline approach^22^							
MP1 (M) – 1st op (Unilat MDmc)	3.91	106.8	106–108	95.5	91–101	− 10.50	10.6
MP2 (F) – 1st op (Unilat MDmc)	3.95	130.8	130–131	123.0	123–123	− 5.93	7.4
MP3 (F) – 2nd op (Unilat MDmc)	4.19	126.6	121–129	121.7	121–123	− 3.90	2.9
MP4 (M) – 1st op (Unilat MDmc)	5.30	118.6	117–120	106.1	106–109	− 10.55	− 1.3
MP5 (M) – 2nd op (Unilat MDmc)	4.30	111.5	109–113	101.4	96–107	− 9.09	− 2.4
MP6 (M) – 1st op (Dorsal midline thalamus)	3.91	101.3	100–104	97.5	93–103	− 3.70	8.30
Unilateral orbitofrontal and ventrolateral prefrontal cortex ablation^22^							
MP1 (M) – 2nd op	4.02	116.6	115–118	116.0	115–117	− 0.51	− 1.5
MP2 (F) – 2nd op	4.08	127.7	127–129	127.0	126–128	− 0.55	− 1.3
MP3 (F) – 1st op	4.06	112.8	112–114	109.9	105–112	− 2.54	0.0
MP4 (M) – 2nd op	5.46	93.5	93–97	92.9	92–94	− 0.53	− 1.9
MP5 (M) – 1st op	4.20	106.6	105–108	108.4	108–109	1.64	0.0
MP6 (M) – 2nd op	4.04	106.1	105–107	105.1	104–106	− 0.95	0.0
RSC ablations using similar midline approach^73^							
RSC1 (M)	3.84	132.6	132–134	130.0	128–132	− 1.98	2.2
RSC2 (M)	3.50	139.3	136–141	136.8	135–140	− 1.80	0.0
RSC3 (M)	3.68	130.4	127–133	131.2	129–133	0.63	0.0
RSC4 (M)	3.55	120.3	120–121	122.4	122–123	1.80	13.6
RSC5 (M)	4.93	144.6	142–147	139.7	137–143	− 3.40	4.6
Fornix transection using similar midline approach^74^							
FX1 (M)	4.98	122.3	121–124	122.7	121–125	0.38	6.0
FX2 (M)	4.79	104.3	103–105	102.4	100–105	− 1.80	4.2
FX3 (M)	4.58	104.3	102–107	105.9	103–107	1.56	− 4.3
FX4 (M)	4.71	143.1	140–150	134.8	134–137	− 5.85	− 10.7

Abbreviations:* Bilateral MDmc and/or MDpc lesions published in^24^; ^#^ Bilateral MDmc lesions published in^23^; ^$^ unpublished data from monkeys with bilateral MDmc lesions. MP1–MP6 listed under unilateral MDmc/midline lesion using midline approach^22^ and unilateral orbitofrontal and ventrolateral prefrontal cortex ablation^22^ are the same animals. The brain manipulations occurred on separate occasions at least three months apart. 1st op means first neurosurgery and 2nd op means second neurosurgery.

On the other hand, the correlation of percent change in sevoflurane concentration and brain manipulation was not significant (*p* = 0.564).

The mean percent heart rate difference scores for the bilateral MD cytotoxin group (Mean (M) = − 6.04, SD = 2.31) and the unilateral MD cytotoxin group (M = − 7.99, SD = 2.95) were negative, indicating a reduction in heart rate after MD manipulation, and were also much larger than the control lesion group (M = − 0.93, SD = 2.07; see Fig. 2A) showing that neuronal loss in the MD substantially affects heart rate. We ran an ANCOVA using the mean percent heart rate difference score as dependent measure and brain manipulation (Group: bilateral MD cytotoxin; unilateral MD cytotoxin; control lesion) as the between groups (fixed) factor, together with the covariate (the monkey’s age in years at time of surgery). This showed a significant effect of Group (*F* (2,25) = 23.45, *p* < 0.001), with an effect size of 0.65, indicating that 65% of the variance was explained by the brain manipulation. The planned Bonferroni *posthoc* comparisons of mean percent heart rate difference scores indicated that, compared to the control lesion group, the heart rate difference score was significantly larger for monkeys in the bilateral MD cytotoxin group (*p* = 0.005) and the unilateral MD cytotoxin group (*p* < 0.001). There was also a difference between bilateral MD cytotoxin and unilateral MD cytotoxin (*p* = 0.042), indicating that the greatest heart rate reduction in the MD-manipulated groups was driven by the unilateral MD cytotoxin monkeys. Thus, manipulations to the MD caused substantial changes in heart rate that were not observed in the animals undergoing control lesion brain manipulations (see Fig. 2A). As expected, age as a covariate also produced an effect, (*F* (1,25) = 7.48, *p* = 0.011). The effect size was 0.23, indicating that 23% of the variance was explained by the age of the monkey at the time of the lesion.

No changes in mean arterial blood pressure, core body temperature, or respiration rate occurred as a consequence of the brain manipulation in any of the surgical groups (*F*’s < 1.0). Note, that throughout surgery, each animal’s body temperature was maintained within the normal range with the use of a warm air blanket and blankets. Additionally, each animal was intubated and mechanically ventilated throughout the neurosurgery under the guidance of the veterinarian anaesthetist (see Materials and Methods).

## Discussion

In the present study, we found that discrete brain manipulations of the MD were associated with variable and observable changes in cardiac output of macaque monkeys: while targeted microstimulation of both the magnocellular and parvocellular subdivisions caused a significant increase in HR and BP, lesions of these same structures using small infusions of cytotoxins led to significant decreases in HR. In contrast, animals that underwent neurosurgery involving cortical ablations or fornix transection following similar surgical procedures and anaesthesia conditions did not show these cardiovascular changes, suggesting that the MD could be an important node in HR modulation, as part of the broader group of brain structures within the central autonomic network^7^.

In our analyses, we tested for a confound of the gaseous anaesthetics, given that both, isoflurane and sevoflurane, are known to cause vasodilation and a reduction in mean arterial pressure which, in turn, elicits a compensatory increase in heart rate in normal healthy humans^27^ and macaque monkeys^28^. However, including the percent change in sevoflurane concentrations as a covariate in the neurosurgical brain manipulation data analysis did not result in a significant effect. For the microstimulation studies, we used isoflurane to have a comparable methodology to previous functional MRI studies in monkeys conducted under general anaesthesia^29–31^. Throughout these experiments, we maintained the isoflurane within the range optimal for experimental purposes, which is lower than the predicted minimum alveolar concentration (MAC) value for macaque monkeys (1.3%;^29,32^; see Fig. 1). However, at times and following veterinary instructions, it was necessary to increase the isoflurane concentration to ensure adequate depth of anaesthesia was maintained, particularly during microstimulation periods (see Fig. 1). Thus, it is not surprising that the linear mixed-effects models showed that changes in isoflurane levels were associated with changes in HR. Nevertheless, microstimulation of both subdivisions of the MD quickly led to a clear increase in HR from the onset (see Fig. 1), before any increase in isoflurane levels.

Intriguingly, the increases in heart rate we observed with microstimulation did not cause an obvious change in the depth of anaesthesia (i.e., increased muscle tone or spontaneous respiratory drive), although, as mentioned above, the concentration of isoflurane was also usually increased as a consequence of the increase in HR change at these times. At least one other primate study has shown similar results, whereby microstimulation of the MD, while the monkeys were sleeping naturally, did not cause them to awaken^33^. This is in marked contrast to other studies that document that monkeys awaken from natural sleep or induced anaesthesia after microstimulation in either the centrolateral thalamic nuclei or other intralaminar thalamic nuclei that are adjacent to the MD^34–36^, but are interconnected to different brain circuits (e.g., the reticular activating system;^37^) or after microstimulation involving both the MD and intralaminar thalamic nuclei^38^.

**Figure 3:**
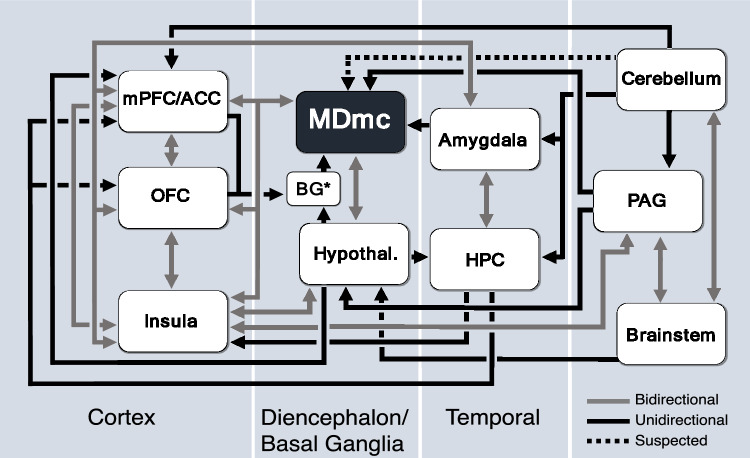
Schematic diagram outlining the brain structures implicated in cardiovascular control in response to physiological or emotional stimuli. The structures within this circuit are highly interconnected providing an effective basis for the integration of multiple information streams necessary for cognitive or emotional processing. Furthermore, the magnocellular subdivision of the mediodorsal thalamus (MDmc) with its diverse array of inputs and reciprocal connections to the frontal lobes and insular cortex appears to be well positioned as an important node within this extended neural circuitry. The main (not exhaustive) connections of this circuit are indicated with directional arrows. Reciprocal connections are in grey with double arrowheads and unidirectional connections are in black with a single arrowhead. Connections that require further verification are shown as dashed black lines with a single arrowhead. The larger blue-gray boxes group each structure into the broader category of the brain region it belongs to. *Not all the basal ganglia (BG) connections are indicated. Abbreviations: ACC, anterior cingulate cortex; HPC, hippocampus; Hypothal., hypothalamus; mPFC, medial prefrontal cortex; OFC, orbitofrontal cortex; PAG, periaqueductal gray.

Of course, we cannot rule out that some of our microstimulation targeting the MD did not also affect the adjacent midline and intralaminar thalamic nuclei, although previous microstimulation studies using the same technique showed the effect of microstimulation to be highly local^39,40^. Thus, the absence of awakening despite the substantial changes in heart rate we observed and the neuroanatomical connectivity of the MD, suggest that the functional role of MD nuclei, and particularly the MDmc subdivision (see Fig. 3) are more aligned to the limbic circuitry than to the ascending reticular activating system^37^. For now, given our findings are only observational, it is not possible to confirm which output from the MD is linked to the HR changes. However, as indicated in Fig. 3, the MD appears to be a functional node in the central autonomic network with many diverse inputs from the amygdala, periaqueductal gray, and lateral hypothalamus, as well as from midbrain and brainstem limbic circuit structures and the locus coeruleus^11–13,41,42^. The reciprocal connectivity with the frontal lobes in combination with these subcortical inputs further suggest the MD functional contribution is related to cognition and executive functioning as a consequence of interoceptive physiological signalling rather than general arousal^43,44^.

Nevertheless, the MD has been observed to contribute to sleep, as neuropathology and positron emission tomography of people diagnosed with fatal familial insomnia indicates the MD is particularly affected^45^. Furthermore, a recent neurophysiology study in rats demonstrated suppression of neural activity in the MD associated with hippocampal ripples^46^. Hippocampal ripples typically occur in NREM sleep^47,48^ and, intriguingly, heart rate and blood pressure are reduced during this sleep stage^49^. Given our current findings, it is possible to hypothesise that this MD activity suppression leads to changes in HR to support the hippocampal interactions with the frontal cortex in generating the appropriate neuronal and physiological activity for optimal learning to occur ‘online’, when rapid integration of task relevant information is required^25,50^, and ‘off-line’, for effective consolidation to occur within the frontal lobes during hippocampal ripples^46^. Supporting this hypothesis, a recent neuroimaging study showed in older adults ‘at-risk’ of dementia that there are functional connectivity changes to parasympathetic regulation during slow wave sleep within both core and broader central autonomic network brain regions that include the MD^10^. Thus, further deciphering the critical functioning of the MD within this neural circuitry is a must.

Heart rate variability is also affected by respiratory rate, tidal volume, and carbon dioxide concentrations (e.g.,^51,52^). In our observational studies, all animals were intubated and mechanically ventilated to maintain a consistent respiratory rate throughout the entire procedure. In future studies, it will be insightful and important to include measures to assess respiratory rates and other physiological parameters that can determine changes in sympathetic and parasympathetic tone and arousal while animals are awake and completing experiments involving higher cognitive tasks in combination with recording neuronal signals from the MD and interconnected structures of the central autonomic network (see Fig. 3). Nevertheless to exclude the mechanical ventilation as a potential confound in our analysis, we included the separate group of neurosurgical animals that received the same surgical midline approach and similar mechanical ventilation to provide a valid comparison of any heart rate changes. As indicated in our analysis, the HR in these animals with either fornix transection or retrosplenial cortex ablation was not altered as a consequence of the brain manipulation.

To date, one study in awake macaque monkeys making reward-guided decisions with electrophysiology recordings from the orbitofrontal and dorsal anterior cingulate cortex has observed that higher HR facilitated reaction times during responding^53^. Interestingly, our own study of awake macaque monkeys with cytotoxic MDmc lesions making reward-guided decisions under uncertainty indicated that these animals failed to show the expected *increased* latency to respond during exploration trials when switching their responding (see^23^, Fig. 4c), although HR was not directly measured in this experiment. This result, and other work involving NHPs, shows that the MDmc and MDpc contribute to rapid reward-guided learning of complex visuospatial discriminations, value-based decision making, and updating of optimal choices during uncertainty (e.g.,^22–25,54,55^). Further, although the MDmc in NHPs does not have a role in memory retrieval per se^25^, stroke damage to it can cause deficits when interference filled delays are used in a forced-choice recognition memory task^56^. Together, this neuroanatomical, physiological, and cognitive evidence suggests the MD is critical for regulating neuronal activity to and from the frontal lobes and other interconnected brain structures during more complex cognitive tasks that require interoceptive contemplation together with ‘online’ flexible behavioral responding, or during the support of ‘offline’ consolidation and updating^10,46,57,58^.

Interestingly, previous work has shown heart rate changes after manipulations in the MD in other mammals, and the findings support the variability in cardiac changes we observed in our NHPs under different anaesthesia protocols and different manipulations (see Table 1). For example, in rabbits, researchers documented reductions in HR after pharmacological manipulations or permanent lesions of the MD and correlated these changes with performance deficits in a trace eye-blink conditioned experiment^59,60^. Conversely, removal of the GABA inputs to the MD in rats caused an increase in HR^17^, while microstimulation in anesthetised rabbits to the posterior part of the MD caused a reduction in HR^18^. Future empirical research involving NHPs is vital to provide cross-species comparative models with translational value for humans in the form of similar cardiac responses, similar behavioral phenotypes during task performance (cf. freezing in a rodent context conditioning task versus the avoidance response in a primate human intruder task; see^20^), and comparative human and primate neuroanatomical circuitry^61^.

Some of the neural structures and circuitry involved in cardiovascular functions are represented by interactions within the agranular frontal cortex, common across all species^61,62^. However, these interactions extend into structures of the granular cortex (e.g., orbitofrontal cortex areas 11 and 12 and the frontopolar cortex) that are distinct in humans with homologous regions in primates that are largely absent in rodents^63^. Further, NHPs show a distribution of neuromodulators, like dopamine and norepinephrine, in the MD and other midline thalamic structures that is comparable to humans and shows differences in distribution patterns and innervation in comparison to rodents^64–67^. As further examples of these human-NHP neuroanatomical and neurophysiological similarities, the primate progressive model of Parkinson’s disease has also identified reduced density of dopamine transporter-immunoreactive axons in the MD at the earliest stages of treatment that further decrease when the monkeys become parkinsonian^68^.

NHP models remain essential to understand the brain in normal functioning and allow us to investigate aspects of abnormal functioning linked to neuropsychiatric and neurodegenerative diseases^69^. In addition, further neuroanatomy work in humans and NHPs is essential to better understand the connectivity of the white matter fibre pathways^70^ linking the thalamus, the cortex, other subcortical structures, and the gut.

Our observational results reported here indicate the extent of the HR changes after experimental manipulations to the MDmc and MDpc. Further research must determine this physiological contribution of the MD in addition to its role in cognitive processes. We suggest the MDmc and MDpc could act as nodal structures linking cognitive, limbic, and autonomic circuits, with interdependent pathways running via the MD to and from the cortex and, importantly, being viable targets for future treatment strategies in many brain diseases and disorders. For this future work, the use of NHPs (and other relevant animal models) will be crucial for the development of any such viable treatments^71^.

## Methods and materials

### Subjects

The microstimulation experiments involved three male rhesus macaques (*Macaca mulatta*) monkeys (mean age: 6.8 years old, mean weight 10.75 kg) and the neurosurgical experiments involved twenty-four rhesus macaques (2 females) (see Table 1 for the individual monkey’s data). All the monkeys were socially housed within same-sex groups (between 2–5 animals), with a 12 h light/dark cycle and always had ad libitum access to water. Food was provided at scheduled times throughout the daylight hours. All experimental procedures were performed in compliance with the United Kingdom Animals (Scientific Procedures) Act of 1986. The Home Office (UK) approved and licensed all procedures after extensive review by the University of Oxford Animal Care and Ethical Review Committee, the sub-committee of NHP Home Office Inspectors, and the independent advisory group, the Animal Sciences Committee. The housing and husbandry compiled with the European Directive (2010/63/EU) for the care and use of laboratory animals. All reporting of animals and experiments followed the recommendations detailed in the ARRIVE guidelines.

### Anaesthesia

Each monkey was sedated prior to each manipulation using an intramuscular injection of ketamine (7.5 mg/kg) combined with midazolam (0.1 mg/kg) and medetomidine (0.02 mg/kg). Once sedated, a nonsteroidal anti-inflammatory drug (meloxicam, 0.2 mg/ kg) for analgesia, and an H2 receptor antagonist (ranitidine, 1 mg/kg) to protect against gastric ulceration as a side effect of nonsteroidal anti-inflammatory drug treatment were administered via intravenous (IV) route. An intravenous cannula was used for delivery of fluids (sterile Hartmann’s solution, 2 ml/kg/hr) throughout each procedure. Normal body temperature was maintained using blankets, bubble wrap, and heated wheat bags.

Monkeys were intubated, ventilated with intermittent positive pressure to ensure a constant respiration rate during the functional fMRI scan, and maintained on isoflurane anaesthesia for the microstimulation-fMRI experiments^31,72^, or sevoflurane anaesthesia for all of the neurosurgeries^22–24,73,74^. The concentration of isoflurane ranged from 0.9 to 2.5% (mode = 1.0%; delivered in a 50:50 mixture of medical air and oxygen) to maintain adequate depth of anaesthesia during the functional scans, in accordance with veterinary instructions. The concentrations of sevoflurane ranged from 1.4% to 3.55%, mixed with medical oxygen. During neurosurgery, some monkeys also received either a continuous IV infusion of the injectable anaesthetic Propofol (0.05 mg/kg/min), or an opioid, alfentanil (0.02 mg/kg/hr). All monkeys undergoing neurosurgery also received bolus IV infusions of buprenorphine (0.01 mg/kg), every 4–6 h. Respiration rate (maintained at 15–18 breathes per minute), inspired and expired CO2, and inspired and expired isoflurane or sevoflurane concentrations were monitored using a VitalStore Monitor (Vetronic Services). Heart rate was measured throughout using a pulse oximeter (Nonin, Nonin Medical Inc.) and blood pressure was measured via an oscillometric non-invasive method (Sentinel Blood Pressure Monitor, Vetronic Services). Together with core temperature, these parameters were recorded manually every five minutes throughout the scans or neurosurgical procedures.

### Microstimulation of the mediodorsal nucleus

As part of a larger study involving the simultaneous acquisition of functional magnetic resonance images (fMRI) with the animal under general anaesthesia, we microstimulated the right MDmc of a male macaque monkey (M1), the right MDpc of a second macaque monkey (M2), and the right MDmc and MDpc of a third male macaque monkey (M3) using similar procedures to those previously described^39,40^. For the monkey that received microstimulation to both subdivisions of the MD (M3), separate sessions were performed for each, with three months elapsing between sessions. A digital stimulator (World Precision Instruments DS8000) coupled to a current isolator (World Precision Instruments DLS100) was used to deliver a train of 1 mA biphasic pulses at 200 Hz for 250 ms (50 pulses; 0.48 ms pulse duration, 4.52 ms inter-pulse interval) through a platinum-iridium electrode (FHC). The electrode was lowered to the area of interest (MDmc or MDpc) and its position was verified with an anatomical scan (MPRAGE, 0.6*0.6*0.6 mm). A platinum wire resting on the dura served as ground. The pulse train was delivered every 2 s throughout a period of 48 s, for a total of 24 stimulation trains, and this stimulation sequence was alternated with 48 s of no stimulation. This stimulation protocol is needed to elicit activation during functional neuroimaging under general anaesthesia^39^ and was administered for periods lasting between 5 to 90 min (mean duration: 61 min). Stimulation periods were preceded by periods of at least 35 min in which the microelectrode was lowered into the target MD subdivision and allowed to settle before commencing the microstimulation. Equally, throughout the experiment, the microstimulation protocol was stopped at least once for periods between 5 to 90 min of duration (mean: 38 min), as required to assess the animal’s general state. Thus, all experimental sessions had at least two stimulation periods and two periods without stimulation but with the electrode already placed in the stimulation site. Here we compare the heart rate (HR) and mean arterial pressure (BP) between these periods: ‘stimulation periods’ were considered from the moment the microstimulation was started until it was stopped (shaded areas in Fig. 1A), whereas ‘baseline periods’ were considered from the moment the electrode was left in the stimulation site until the beginning of the stimulation. In the case of ‘baseline periods’ between two ‘stimulation periods’, the first ten minutes post-stimulation were not considered in the analyses to discard any potential stimulation aftereffects (non-shaded areas in Fig. 1A).

### Cytotoxin injections to the MD

Fifteen monkeys had MDmc or MDpc cytotoxin permanent lesions: six received a unilateral MDmc lesion^22^ and nine received bilateral lesions to the MDmc and/or MDpc. Behavioural results from six of these monkeys with bilateral lesions have been previously published (MD1, MD2:^23^; MDP1, MDP2, MDP3, MDP4:^24^. The injections consisted of a combined mix of ibotenic acid (10 mg/ml) and NMDA (10 mg/ml) via a midline surgical approach as described elsewhere (e.g.,^22,24^). To visualize the dorsal thalamus prior to the injections, it was necessary to create a small hole within the corpus callosum (up to 6 mm using a glass aspirator) and cauterize some of the tela choroidea within the third ventricle to target the thalamus underneath.

### Control group neurosurgical procedures

To have a neurosurgical group of monkeys to compare differences in heart rate changes because of similar invasive brain manipulations under surgical anaesthesia using sevoflurane while being mechanically ventilated, we included data from other groups of monkeys undergoing neurosurgical procedures in our lab.

All these monkeys received identical peri-operative medication and care, and the procedures were performed under aseptic conditions using direct visualization of each target structure with the aid of an operating microscope. Six monkeys received unilateral ventral prefrontal cortical ablations^22^; these six animals also received unilateral MDmc cytotoxin injections in separate surgical procedures, with two months elapsing between each procedure – see Table 1 for details), five monkeys received bilateral ablations to the retrosplenial cortex (RSC1, RSC2, RSC3, RSC4, RSC5:^73^), and four monkeys received a fornix transection (FX1, FX2, FX3, FX4:^74^); these last two groups of monkeys underwent a similar midline surgical approach as used for the neurosurgery to produce cytotoxic injections into the MD. Detailed explanations of the neurosurgical procedures for retrosplenial cortex lesions, ventral prefrontal and orbitofrontal cortex ablations, and fornix transection have been published^22,73,74^.

### Statistical analysis

#### Microstimulation

From the anaesthesia records, we obtained the heart rate (HR), mean arterial pressure (BP), and isoflurane levels during the stimulation and baseline periods and used these parameters to compute linear mixed-effects models separately for each MD subdivision using MATLAB (MathWorks, v. 9.12.0.1884302, R2022a) as follows:

$${\text{X}}\; \sim \;{\text{stimulation}}\;{\text{period}} + {\text{isoflurane}}\;{\text{level}}\; + \left( {1 + {\text{isoflurane}}\;{\text{level|NHP}}\;{\text{ID}}} \right),$$

where X is the dependent variable under study (HR or MP), ‘stimulation’ (stimulation or baseline period) and ‘isoflurane level’ are fixed factors, and the NHP ID is the grouping random effect. Given that the isoflurane levels could potentially differ between each monkey, we included the isoflurane level as a random effect grouped by NHP ID. The level of statistical significance to reject the null hypothesis was alpha = 0.05.

#### Cytotoxin injections and neurosurgical manipulations

Our dependent variable, the percent heart rate difference score was calculated as,

$$\left(\frac{\overline{x }\, procedure-\overline{x} \,baseline }{\overline{x}\, baseline }\right) \times 100$$

where $\overline{x } procedure$ is the mean heart rate during the MD manipulation procedure (first 40 min of cytotoxin infusions into the MD) and $\overline{x } baseline$ is the heart rate during the 40 min before the MD manipulation following the removal of the bone flap, with the animal under general anaesthesia using sevoflurane (see % change of volatile agent for each individual animal in Table 1). Mean arterial blood pressure changes during neurosurgery were calculated using the same formula.

Other factors (e.g., underlying co-morbidities) can also affect changes in heart rate. Thus, in our analysis, we excluded two male monkeys who had also received MDmc bilateral lesions but had known underlying health problems (not shown in Table 1): one had dysrhythmia, which was recorded during its neurosurgery using an electrocardiogram, and the other had chronic diarrhoea and was prescribed additional daily medication to manage the condition.

The data analyses were completed in SPSS version 28. Pearson’s correlation coefficient analyses were used to characterize any relationship between the monkey’s age at the time of surgery or sevoflurane concentrations and the mean percent heart rate difference score. A univariate analysis of covariance (ANCOVA) was used to assess the impact of the different neurosurgical manipulations on the dependent variable (mean percent heart rate difference score), with the monkey’s age at the time of surgery as a covariate. Bonferroni post hoc comparisons were used to determine any significant interactions between the groups.

## Data Availability

All data analysed during this study are included in this article.
